# IRAK-M Regulates Chromatin Remodeling in Lung Macrophages during Experimental Sepsis

**DOI:** 10.1371/journal.pone.0011145

**Published:** 2010-06-16

**Authors:** Kenneth Lyn-Kew, Eric Rich, Xianying Zeng, Haitao Wen, Steven L. Kunkel, Michael W. Newstead, Urvashi Bhan, Theodore J. Standiford

**Affiliations:** 1 Division of Pulmonary and Critical Care Medicine, Department of Medicine, The University of Michigan Medical School, Ann Arbor, Michigan, United States of America; 2 Department of Pathology, The University of Michigan Medical School, Ann Arbor, Michigan, United States of America; Louisiana State University, United States of America

## Abstract

Sepsis results in a profound state of immunosuppression, which is temporally associated with impaired leukocyte function. The mechanism of leukocyte reprogramming in sepsis is incompletely understood. In this study, we explored mechanisms contributing to dysregulated inflammatory cytokine expression by pulmonary macrophages during experimental sepsis. Pulmonary macrophages (PM) recovered from the lungs of mice undergoing cecal ligation and puncture (CLP) display transiently reduced expression of some, but not all innate genes in response to LPS. Impaired expression of TNF-α and iNOS was associated with reduced acetylation and methylation of specific histones (AcH4 and H3K4me3) and reduced binding of RNA polymerase II to the promoters of these genes. Transient impairment in LPS-induced cytokine responses in septic PM temporally correlated with induction of IRAK-M mRNA and protein, which occurred in a MyD88-dependent fashion. PM isolated from IRAK-M^−/−^ mice were largely refractory to CLP-induced impairment in cytokine expression, chromatin remodeling, recruitment of RNA polymerase II, and induction of histone deacetylase-2 observed during sepsis. Our findings indicate that systemic sepsis induces epigenetic silencing of cytokine gene expression in lung macrophages, and IRAK-M appears to be a critical mediator of this response.

## Introduction

Sepsis results in profound suppression of both innate and acquired immune responses [1], [2], [3]. This syndrome influences the effector activity of a variety of immune cells, including blood monocytes, tissue macrophages, neutrophils, dendritic cells, and lymphocytic populations [2], [4], [5], [6], [7]. Alterations in monocyte/macrophage function during sepsis include impaired responsiveness to LPS and other pathogen associated molecular patterns (PAMPs), resulting in reduced inflammatory cytokine production, adhesion molecule expression, respiratory burst, and production of reactive nitrogen species [5], [6], [7], [8], [9], [10], [11]. The reprogramming of effector responses in septic macrophages resemble but are not identical to that observed in endotoxin-tolerized macrophages, whereby initial exposure to LPS renders cells refractory to a subsequent LPS challenge. Phenotypic changes in macrophages during sepsis are often transient, and failure to reverse this deactivated state has been associated with worse clinical outcomes in sepsis patients [5], [7], [10], [11].

The mechanisms accounting for sepsis-induced deactivation and endotoxin tolerance are incompletely defined, but are characterized by impaired toll-like receptor (TLR) mediated activation of both NF-κB- and mitogen-activated protein kinase (MAPK)-dependent genes [5], [9], [10]. Interleukin-1 receptor-associated kinase-M (IRAK-M; also referred to as IRAK-3) is a protein in the IRAK family that has been shown to be an important negative regulator of TLR-mediated cell signaling [12], [13], [14]. In contrast to IRAK-1 and IRAK-4, IRAK-M lacks kinase activity and negatively regulates signaling through MyD88-dependent TLRs [14]. This protein has been shown to regulate critical aspects of innate immunity, including the development of endotoxin tolerance and sepsis-induced alterations of antimicrobial responses [14], [15], [16], [17], [18]. IRAK-M was initially believed to be limited to cells of monocytic lineage. However, we and others have shown that cells of both hematopoetic and non-hematopoetic origin express biologically active IRAK-M [1], [18], [19], [20]. The role of this inhibitory protein in regulating key aspects of cytokine gene expression during sepsis has been incompletely defined.

Gene expression is not only dictated by the genetic code, but also by epigenetic alterations of chromatin that can substantially modify gene expression in either an inheritable or transient fashion [21]. For example, methylation of cysteine residues within CpG islands of promoter regions can function to suppress gene activation [22]. Moreover, post-translational chemical modification of histones binding to promoter regions of DNA regulate access of DNA to transcription factors, regulatory proteins or RNA polymerase required for gene transcription [23], [24]. Examples include acetylation of histone 4 or trimethylation of lysine 4 of histone 3 (H3K4me3) generally enhances access to DNA leading to gene activation, whereas di-or tri-methylation of lysine 27 of histone 3 closes DNA resulting in a gene silencing effect [24], [25]. Endotoxin tolerance has been associated with remodeling of chromatin in the promoter region of several tolerizable genes [26], [27]. Likewise, a decrease in activating histone PTMs (i.e. H3K4me3) and concomitant increase in suppressive PTMs (H3K27me3) has been noted in dendritic cells isolated from mice out to 6 weeks after induction of sepsis [28]. The contribution of histone PTMs to altered monocyte/macrophage effector responses in sepsis have not been described.

In this study, we investigated the role of IRAK-M in regulating lung macrophage effector responses during sepsis, and determined if this key protein might regulate gene expression by altering critical histone PTMs in septic lung macrophages.

## Methods

### Ethical Statement

All animals were handled in strict accordance with good animal practice as defined by the relevant national and/or local animal welfare bodies, and all animal work was approved by University Committee on Use and Care of Animals at the University of Michigan (UCUCA approval number 09721).

### Mice

Colonies of IRAK-M^−/−^ and MyD88^−/−^ mice bred on a B6 background were established at the University of Michigan [15]. Age- and sex-matched specific pathogen–free 5- to 8-week-old C57BL/6J mice were purchased from the Jackson Laboratory (Bar Harbor, Maine). All animals were housed in specific pathogen–free conditions within the University of Michigan animal care facility (Ann Arbor, Michigan, USA) until the day of sacrifice. All animal experiments were performed in accordance with NIH policies on the humane care and use of laboratory animals and approved by the University Committee on Use and Care of Animals (UCUCA) at the University of Michigan.

### Cecal ligation and puncture (CLP)

CLP was used as a model of systemic sepsis syndrome as previously described [8], [15]. Pathogen-free C57BL/6 mice were anesthetized with a mixture of i.p. xylazine and ketamine. A 1-cm longitudinal incision was made to the lower-right quadrant of the abdomen and the cecum exposed. The distal one-third of the cecum was ligated with a 3-0 silk suture and punctured through with a 26-gauge needle. Puncture with this gauge needle results in a marked septic response but with death occurring in 0–10% of animals. The cecum was then placed back in the peritoneal cavity, and the incision was closed with surgical staples. In sham surgical controls, the cecum was exposed but not ligated or punctured, then returned to the abdominal cavity. All mice were administered 1 ml of sterile saline s.c. for fluid resuscitation during the immediate postoperative period.

### Total lung leukocyte preparation by lung digest

Lungs were removed from euthanized animals and leukocytes prepared as previously described [15]. Briefly, lungs were minced with scissors to fine slurry in 15 ml of digestion buffer (RPMI, 10% fetal calf serum, 1 mg/ml collagenase [Roche Diagnostics], 30 µg/ml DNase [Sigma-Aldrich]) per lung. Lung slurries were enzymatically digested for 30 minutes at 37°C. Any undigested fragments were further dispersed by drawing the solution up and down through the bore of a 10-ml syringe. The total lung cell suspension was pelleted, resuspended, and spun through a 40% Percoll gradient to enrich for leukocytes. Cell counts and viability were determined using Trypan blue exclusion counting on a hemacytometer. Cytospin slides were prepared and stained with a modified Wright-Giemsa stain. Cells were then cultured in RPMI with or without 100 ng/mL LPS (Sigma-Aldrich, USA). Adherent PM were washed and lysed for RNA isolation. Confirmation of purity of macrophage cultures was determined by flow cytometry using light scatter characteristics and expression of CD45 and CD11b. Cells were >95% macrophages using this isolation technique.

### Real-time quantitative RT-PCR

Measurement of gene expression was performed utilizing the ABI Prism 7000 Sequence Detection System (Applied Biosystems) as previously described [15]. Briefly, the primers and probe for β-actin was designed using Primer Express software (Applied Biosystems). The primers, placed in different exons, were tested to ensure that they do not amplify genomic DNA. Primers and probe nucleotide sequences for mTNF-α were as follows: forward 5′-CAGCCGATGGGTTGTACCTT-3′, reverse 5′-TGTGGGTGAGGAGCACGTAGT-3′, probe 5′-TCCCAGGTTCTCTTCAAGGGACAAGGC-3′; for mIL-12p40: forward 5′-AGACCCTGCCCATTGAACTG-3′, reverse 5′-GAAGCTGGTGCTGTAGTTCTCATATT-3′, probe 5′-CGTTGGAAGCACGGCAGCAGAA-3′; for mIRAK-M: forward 5′-TGAGCAACGGGACGCTTT-3′, reverse 5′-GATTCGAACGTGCCAGGAA-3′, probe 5′-TTACAGTGCACAAATGGCACAACCCC-3′; miNOS forward, 5′-CCC TCC TGA TCT TGT GTT GGA-3′; reverse, 5′-CAA CCC GAG CTC CTG GAA-3′; and probe, 5′-TGA CCA TGG AGC ATC CCA AGT ACG AGT-3′; for mLcn2 forward, 5′-GGGCAGGTGGTACGTTGTG-3′; reverse, 5′-CAT CGT AAA GCT GCC TTC TGTTT-3′; and probe, 5′-CCTGGCAGGCAATGCGGTCC-3′;for mHDAC-1 forward 5′-CTGGGAGGAGGTGGCTACAC-3′, reverse 5′-GCCACCGCTGTTTCGTAAGT-3′, and probe 5′-ATCCGGAATGTTGCTCGCTGCTG-3′; for mHDAC-2: forward 5′-GAAGATTGTCCGGTGTTTGATG-3′, reverse 5″-CACAGCCCCAGCAACTGAA-3′, and probe 5′-ACTCTTTGAGTTTTGTCAGCTCTCCACGGG-3′ for mHDAC-3: forward 5′-CCCAGTGTCCAGATTCATGATG-3′, reverse 5′-GGCCTCGTCAGTCCTGTCA-3′, and probe 5′-CCCGGCAGACCTCCTGACGT-3′ for mHDAC-7: forward 5′-CTTTCCCTTGCGTAAAACAGTGT-3′, reverse 5′-GCGTCTCTCCAGGGATTTCTT, and probe 5′-CCCAACCTGAAGTTGCGCTACAAACC-3′; for mβ-actin: forward 5′-CCGTGAAAAGATGACCCAGATC-3′, reverse 5′-CACAGCCTGGATGGCTACGT-3′, probe 5′-TTTGAGACCTTCAACACCCCAGCCA-3′. Specific thermal cycling parameters used with the TaqMan One-Step RT-PCR Master Mix Reagents kit (Applied Biosystems, USA) included 30 minutes at 48°C, 10 minutes at 95°C, and 40 cycles involving denaturation at 95°C for 15 seconds, annealing/extension at 60°C for 1 minute. Relative quantitation of cytokine mRNA levels was plotted as fold change relative to untreated control cells of the lung. All experiments were performed in triplicate.

### Chromatin Immunoprecipitation Assay (ChIP)

Lung macrophages were isolated as described above. The chromatin immunoprecipitation (ChIP) procedure was performed using a modification of an assay kit (Upstate Biotechnology, Charlottesville,VA) according to the manufacturer's instructions [28]. Briefly, 2–3×10^6^ isolated PM were treated with vehicle or LPS 1 µg/mL for 2–4 hours. DNA-protein structure was then cross-linked by 1% formaldehyde for 10 minutes at 37°C. Cells were collected and lysed in 400 µL SDS lysis buffer. The resulting lysates were sonicated to obtain DNA fragments ranging from 200 to 1000 bp (base pairs) using a Branson Sonifier 450 (VWR, West Chester, PA) six times for periods of 15 seconds each. After centrifuging, the supernatant containing chromatin was diluted, and an aliquot (2% volume) was saved to indicate the input DNA in each sample. The remaining chromatin fractions were pre-cleared with salmon sperm DNA/protein A agarose beads followed by immunoprecipitation with the following antibodies: anti-acetyl histone H4 (Upstate Biotechnology), anti-H3K4me3 (Upstate), or anti-RNA polymerase II (Santa Cruz Biotechnology) overnight at 4°C with gentle rotation. Cross-linking was reversed for 4 hours at 65°C and was followed by proteinase K digestion. DNA was purified by standard phenol/chloroform and ethanol precipitation and was subjected to real-time PCR. Primers for mTNF-α promoter are as follows: forward, GGAAATAGACACAGGCATGGTCT; reverse, CCTACACCTCTGTCTCGGTTTCTT; iNOS promoter: forward, CCTCCCCAGAACTTATTGCAAG; reverse GAAAAT CCCCCTAGCACATCCT. Data was expressed as percent of input DNA or fold increase over unstimulated cells.

### Immunoflorescence staining for IRAK-M

Lung macrophages were isolated as described above. The cells were permeablized with a 1∶1 mixture of methanol and acetone. After washing with PBS, anti-IRAK-M antibodies (abcam) were applied and the cells incubated in their presence for one hour. After washing, antibodies against the primary antibody labeled with FITC were applied to the cells and incubated for one hour. The cell were then mounted in DAPI mounting media and viewed on the fluorescent microscope

### Western immunoblotting for HDAC-2 expression

Whole cell lysates were obtained by treating cells with RIPA buffer (1% w/w NP-40, 1% w/v sodium deoxycholate, 0.1% w/v SDS, 0.15 M NaCl, 0.01 M sodium phosphate, 2 mM EDTA, and 50 mM sodium fluoride) plus protease and phosphatase inhibitors. Protein concentrations were determined by the Bio-Rad DC protein assay (Bio-Rad Laboratories, Hercules, CA). Samples were electrophoresed in 4–12% gradient SDS-PAGE gels, transferred to nitrocellulose and blocked with 5% skim milk in PBS. After incubation with primary anti-HDAC-2 Ab (Cell Signalling), blots were incubated with secondary antibody linked to horseradish peroxidase and bands visualized using enhanced chemiluminescence (SuperSignal West Pico Substrate, Pierce Biotechnology, Inc., Rockford, IL).

### Statistical analysis

Statistical significance was determined using the unpaired 2-tailed Student's t test or 1-way ANOVA corrected for multiple comparisons where appropriate. P values less than 0.05 were considered statistically significant. All calculations were performed using the Prism 3.0 software program for Windows (GraphPad Software).

## Results

### Abdominal sepsis results in suppression of certain LPS-induced inflammatory cytokine genes in pulmonary macrophages

**Figure 1:**
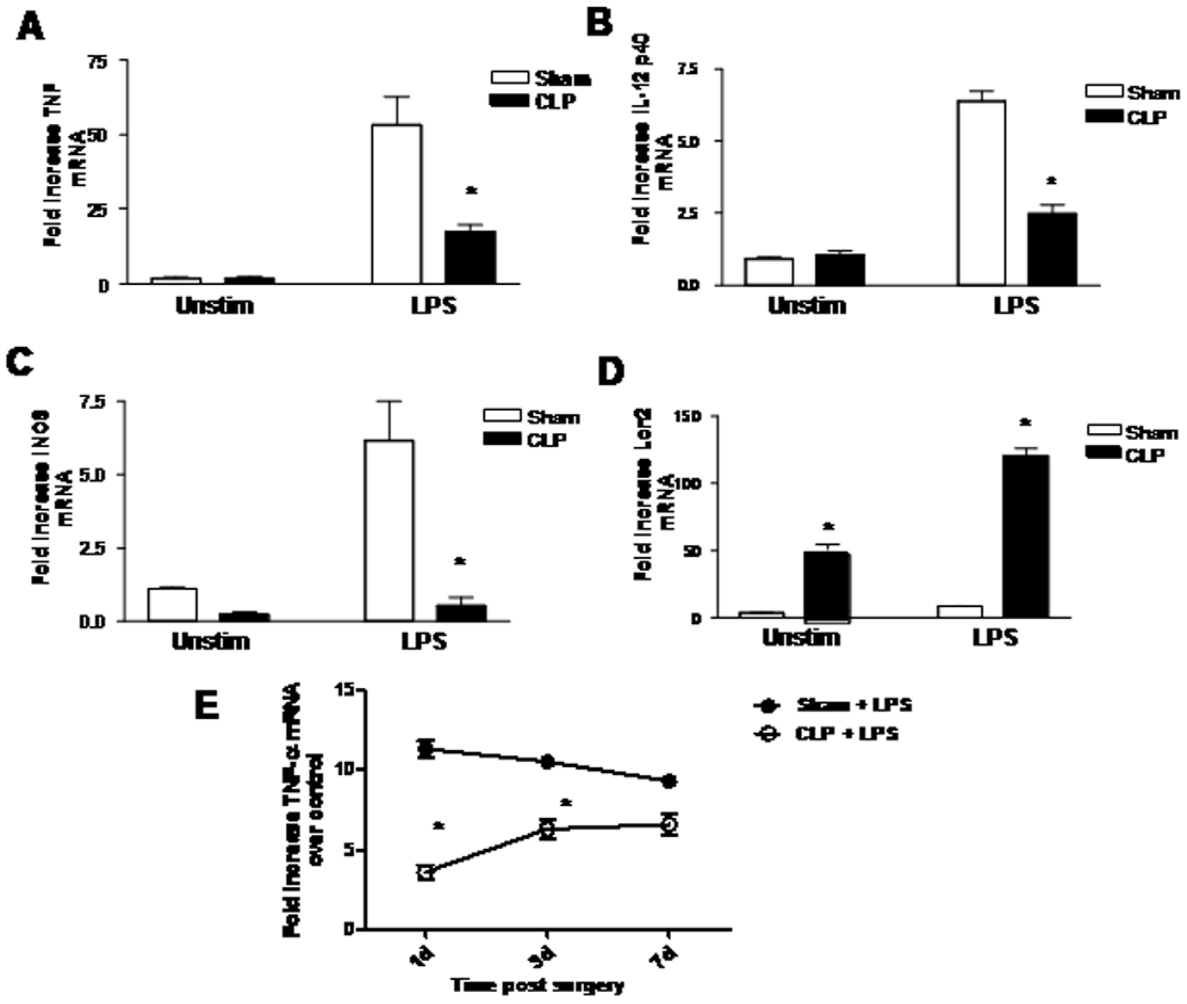
Effect of CLP on ex-vivo cytokine expression by control and LPS-stimulated PM. Cytokine mRNA expression was determined 4 hrs post vehicle or LPS administration by real-time PCR and expressed as fold increase over unstimulated sham PM. Panel E represents TNF-α expression from PM collected at 1, 3, and 7 days post sham surgery or CLP. n = 5 per condition, *p<0.05 as compared to sham counterpart.

We and others have demonstrated impaired expression of inflammatory genes from blood monocytes and tissue macrophages isolated from humans with sepsis and in animal models of sepsis [5], [9], [10], [11], [15]. To further explore the nature of LPS-inducible genes that are dysregulated during the septic response, we induced abdominal sepsis in mice by performing CLP or sham surgery, then isolated pulmonary macrophages (PM) from lung digests 24 hrs after surgery. We chose the 24 hr time point post surgery, as previous studies indicated maximum impairment in innate responses post abdominal sepsis occurred at this time point. Lung macrophages were incubated with LPS (1 µg/ml) or vehicle, then RNA isolated 4 hrs post LPS administration. As shown in Figure 1, minimal constitutive expression of the inflammatory genes TNF-α, IL-12 p40, or iNOS was detected in vehicle treated PM isolated from either sham of CLP animals (panels A-C). Treatment of sham PM with LPS resulted in a substantial increase in the mRNA expression of all of these genes. Importantly, the LPS-induced expression of TNF-α, IL-12p40, and iNOS mRNA by PM isolated from CLP mice was reduced by 67, 73, and >90%, respectively, as compared to LPS-treated sham PM. In contrast, the expression of lipocalin (Lcn2) was enhanced in PM isolated from CLP mice at baseline, as compared to sham PM, and the expression of this gene was further augmented in CLP PM stimulated with LPS (panel D). The results indicate suppression of selected inflammatory genes during sepsis, rather than global suppression of all gene products from macrophages. Moreover, the tolerance phenotype observed in these macrophages was transient, as LPS-induced expression of TNF-α was progressively restored in CLP PM isolated at 3 and 7 days post CLP (panel E).

### Abdominal sepsis results in alteration in activating histone post-translational modifications in lung macrophages

**Figure 2:**
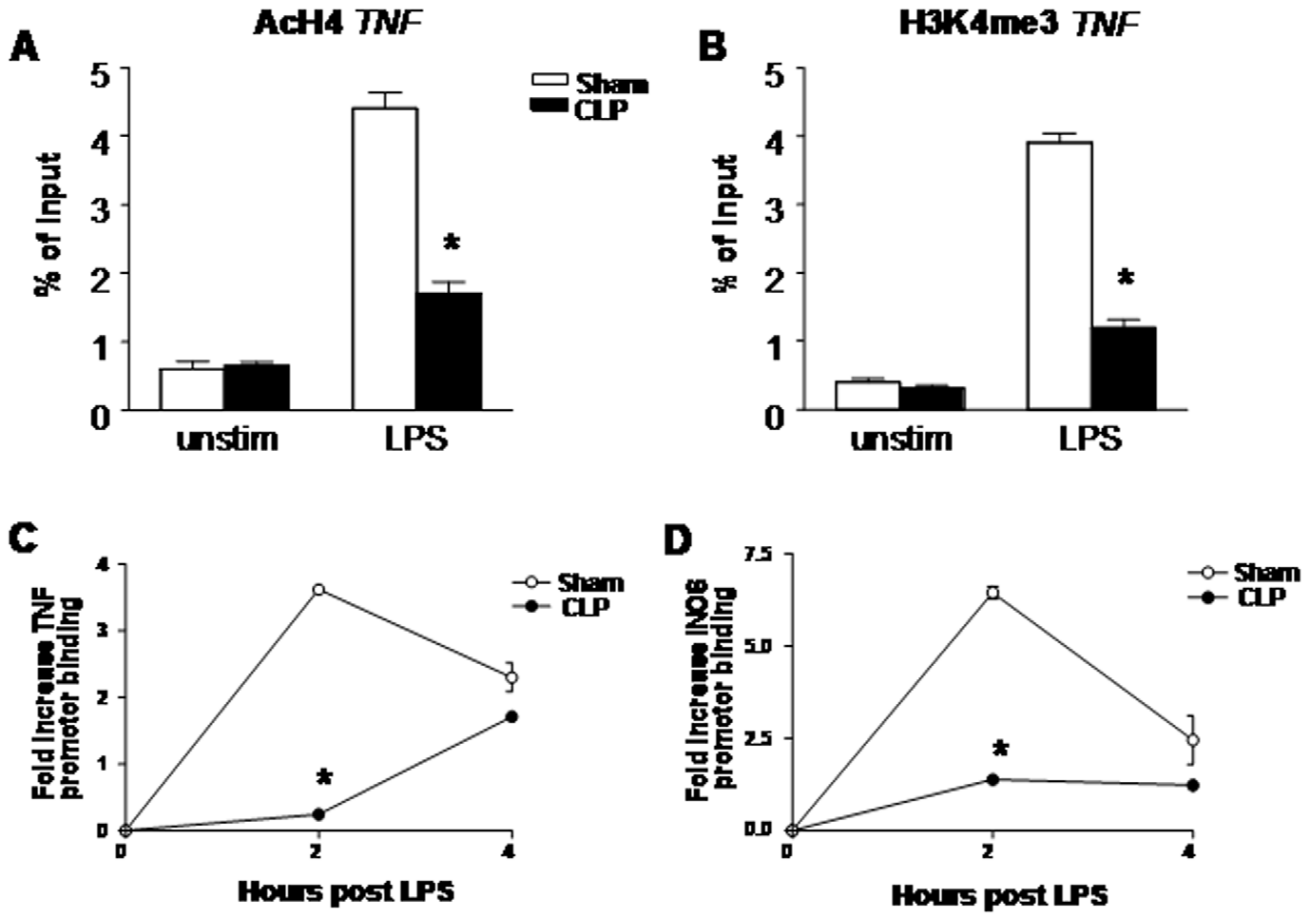
Histone modifications and recruitment of RNA polymerase II to the TNF-α and iNOS promoter. Level of specific histone acetylation (AcH4, panel A) and trimethylation (H3K4me3, panel B) at the TNF-α promoter 2 hrs post LPS or vehicle treatment, as determined by ChIP analysis and expressed as percent of input DNA. Panels C and D are ChIP analyses revealing RNA polymerase II binding to the TNF-α promoter (C) and the iNOS promoter (D) 2 and 4 hrs post LPS, and expressed as fold increase over unstimulated sham cells. Results shown represent mean of 3 separate experiments, *p<0.05 as compared to sham PM.

Post-translational modifications (PTM) of histones binding to the promoters of inflammatory genes can modify the expression of these genes. Two histone PTM generally associated with gene activation include acetylation of histone 4 (AcH4) and trimethylation of histone 3 at lysine-4 [H3K4me3 [24], [25], [26], [28]. To determine if sepsis altered these activating epigenetic marks, PM were isolated from lung digest collected 24 hrs after either sham surgery or CLP, then incubated with vehicle or LPS ex vivo, and acetylation and/or trimethylation of histones binding to the murine TNF-α promoter assessed 2 hrs later by chromatin immunoprecipitation (ChIP) analysis. Treatment of PM isolated from sham-operated mice with LPS resulted in a considerable increase in acetylation of histone 4 and trimethylation of the fourth lysine residue of histone 3 (7.3-fold and 9.8-fold, respectively, Figure 2A). Interestingly, levels of AcH4 and H3K4me3 in LPS-treated PM isolated from CLP mice were significantly reduced compared to sham cells. Because AcH4 and H3K4me3 function to enhance gene transcription, in part, by facilitating binding of RNA polymerase II to promoters of regulated genes, we next assessed binding of RNA polymerase II to the TNF-α promoter at both 2 and 4 hrs post LPS treatment in sham and CLP macrophages. As shown in Figure 2B, LPS induced an increase in binding of RNA polymerase II to the TNF promoter, maximal at 2 hrs post LPS, and declining by 4 hrs. By comparison, binding of RNA polymerase was substantially reduced in CLP macrophages, especially at 2 hrs post LPS.

### Effect of the histone deacytylase inhibitor trichostatin (TSA) on expression of TNF-a from PM post sham surgery or CLP

**Figure 3:**
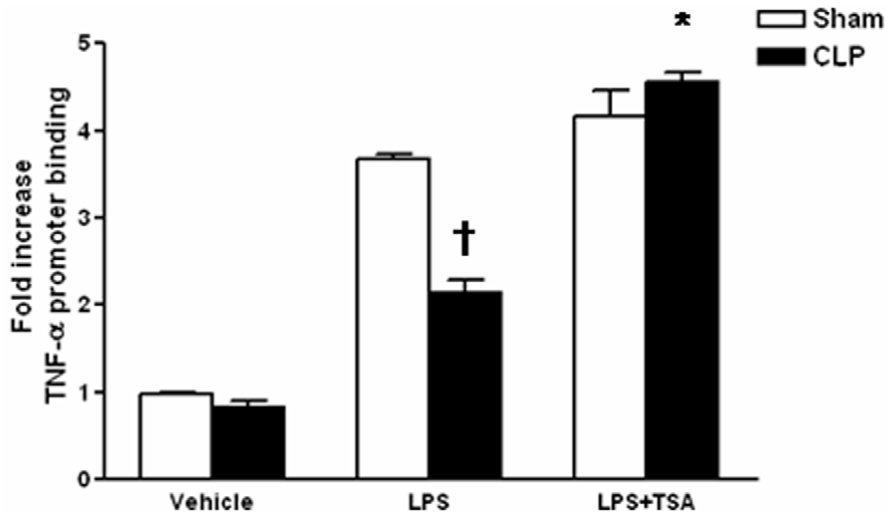
Effect of TSA on H4 acetylation at the TNF-α promoter post CLP. Level of specific histone acetylation (AcH4) at the TNF-α promoter 4 hrs post LPS or vehicle treatment in the presence or absence of TSA, as determined by ChIP analysis and expressed as fold-increase over control. Each condition represents pooled PM from 3 separate mice. †p<0.05 as compared to sham/LPS group, *p<0.05 as compared to CLP/LPS group.

To assess the contribution of histone deacetylases to suppression of macrophage cytokine expression post CLP, we isolated PM from mice 24 hrs post sham surgery or CLP, incubated cells with the histone deacetylase (HDAC) inhibitor TSA (500 nM, Sigma) or vehicle for one hr, followed by administration of LPS (1 µg/ml) or vehicle. Cells were isolated 3 hrs after LPS and assessed for acetylation of H4 at theTNF-α promoter by ChiP analysis. As shown in Figure 3, treatment with LPS resulted in increased AcH4 in sham PM, which was diminished in PM isolated from animals undergoing CLP. The magnitude of AcH4 was modestly but not significantly increased in LPS-treated macrophages treated with TSA, as compared to sham cells treated with vehicle. However, acetylation at H4 was significantly increased in TSA-treated CLP macrophages over that observed in LPS-stimulated CLP PM. Collectively, these data indicate that the reduced levels of H4 acetylation found in macrophages post CLP is due to enhanced histone deacyetylation activity.

### Abdominal sepsis results in the time dependent expression of IRAK-M in lung macrophages

**Figure 4:**
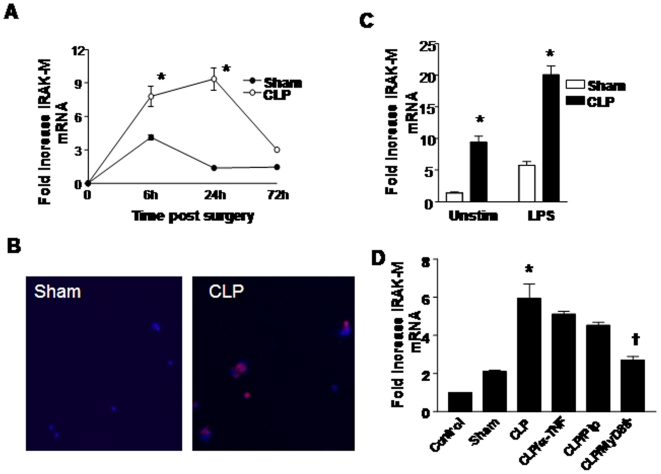
Induction of IRAK-M mRNA and protein after CLP or sham surgery. A. Time dependent expression of IRAK-M after CLP or sham surgery, expressed as fold increase over baseline (time 0) mRNA expression; B. Immunoflourescence demonstrating cell-associated IRAK-M in lung macrophages 24 hrs post surgery; C. Induction of IRAK-M mRNA 24 hrs post sham surgery or CLP in untreated and LPS treated PM ex-vivo; D. Expression of IRAK-M mRNA in PM ex-vivo harvested from sham or CLP animals after treatment in-vivo with anti-TNF antibody or pipericillin (Pip), or in mice deficient in MyD88 (MyD88^−/−^). n =  pooled cells from 3 animals per group, *p<0.05 as compared to sham, †p<0.05 as compared to other CLP groups.

IRAK-M has previously been shown to mediate tolerance induced by several PAMPs, including LPS and peptidoglycan [14], [16]. To determine if IRAK-M was expressed by PM during sepsis, we measured IRAK-M mRNA and protein at various times post CLP or sham surgery. At baseline, lung macrophages expressed minimal amounts of IRAK-M mRNA and protein. When mice were subjected to sham surgery, there was a modest but significant induction of IRAK-M message by 6 hrs post surgery, with mRNA levels returning to baseline by 24 hrs (Figure 4A). By comparison, a greater induction of IRAK-M mRNA was observed in PM obtained from mice undergoing CLP, with a maximal 9-fold increase over baseline observed at 24 hrs, and returning near baseline levels by 72 hrs post CLP. Consistent with induction of IRAK-M mRNA, there was substantial accumulation of intracellular IRAK-M protein in CLP PM at 24 hrs post surgery, with minimal expression noted in PM at any time post sham surgery (panel B). Treatment of PM recovered from sham mice with LPS resulted in a 5-fold increase in IRAK-M mRNA over baseline. However, there was a substantially greater accumulation of IRAK-M message in CLP PM after treatment with LPS, indicating that abdominal sepsis serves to prime cells for augmented expression of IRAK-M in response to a second stimulus (Figure 4, panel C).

The determine if the induction of IRAK-M in PM during abdominal sepsis required endogenous TNF-α or live intact bacteria, mice were pretreated with purified rabbit anti-murine TNF-α antibody (1 mg) i.p. or the broad spectrum antibiotic pipericillin (100 µg i.p.) two hrs prior to surgery, then PM were isolated 24 hrs post sham surgery or CLP. To assess the contribution of the adaptor molecule MyD88, PM were isolated from MyD88^−/−^ mice 24 hrs post CLP. As shown in Figure 4D, the upregulation of IRAK-M mRNA in CLP PM was not significantly altered by treatment with either anti-TNF-α neutralizing antibody or broad spectrum antibiotics. However, the induction of IRAK-M mRNA after CLP was nearly completely abolished in MyD88^−/−^ mice, indicating the requirement for MyD88 in sepsis-induced expression of IRAK-M.

### IRAK-M deficient macrophages are resistant to sepsis-induced suppression of inflammatory genes

**Figure 5:**
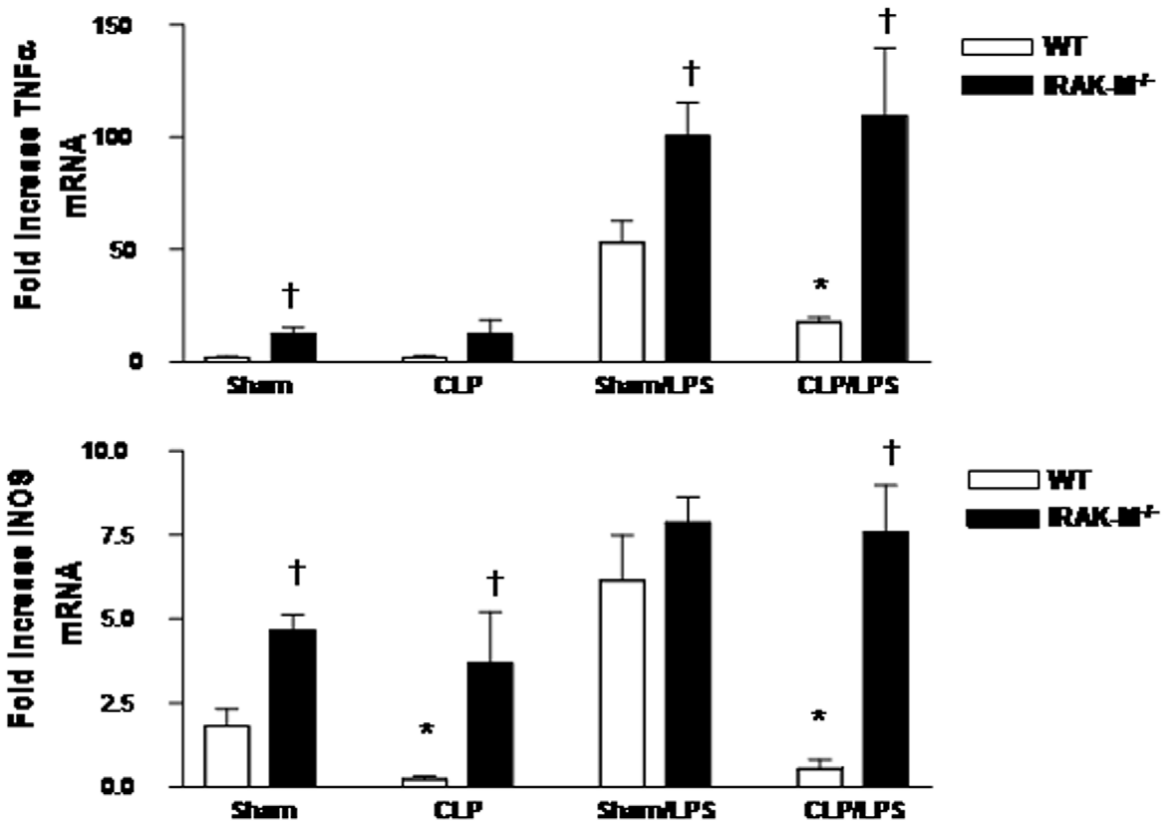
Expression of TNF-α and iNOS mRNA from PM isolated from WT and IRAK-M^−/−^ mice after sham surgery or CLP. PM were isolated 24 hrs post sham or CLP surgery, incubated with vehicle or LPS (1 µg/ml), then mRNA expression determined by real-time PCR 4 hrs later. n = 6 per group, combined from two separate experiments. *p<0.05 as compared to WT sham, †p<0.05 as compared to WT.

To determine if IRAK-M contributed to sepsis-induced suppression of inflammatory genes, we isolated PM from WT and IRAK-M^−/−^ mice 24 hrs after sham surgery or CLP, then assessed for constitutive and LPS-stimulated expression of TNF-α and iNOS mRNA. As observed previously, treatment of sham PM isolated from WT mice with LPS ex vivo resulted in considerable accumulation of TNF-α and iNOS mRNA, which was reduced in PM isolated from WT mice undergoing CLP (Figure 5). The constitutive expression of TNF-α and iNOS was increased in both sham and CLP PM recovered from IRAK-M^−/−^ mice, as compared to WT PM, consistent with the previously noted hyperinflammatory state in IRAK-M deficient macrophages. Importantly, the LPS-induced upregulation of both TNF-α and iNOS observed in sham PM was not significantly diminished in IRAK-M^−/−^ macrophages recovered 24 hrs post CLP, indicating that in the absence of IRAK-M, these cells were resistant to the development of a “tolerized” phenotype during sepsis.

### IRAK-M deficient macrophages are relatively resistant to sepsis-induced suppression of histone PTM and RNA polymerase II binding

**Figure 6:**
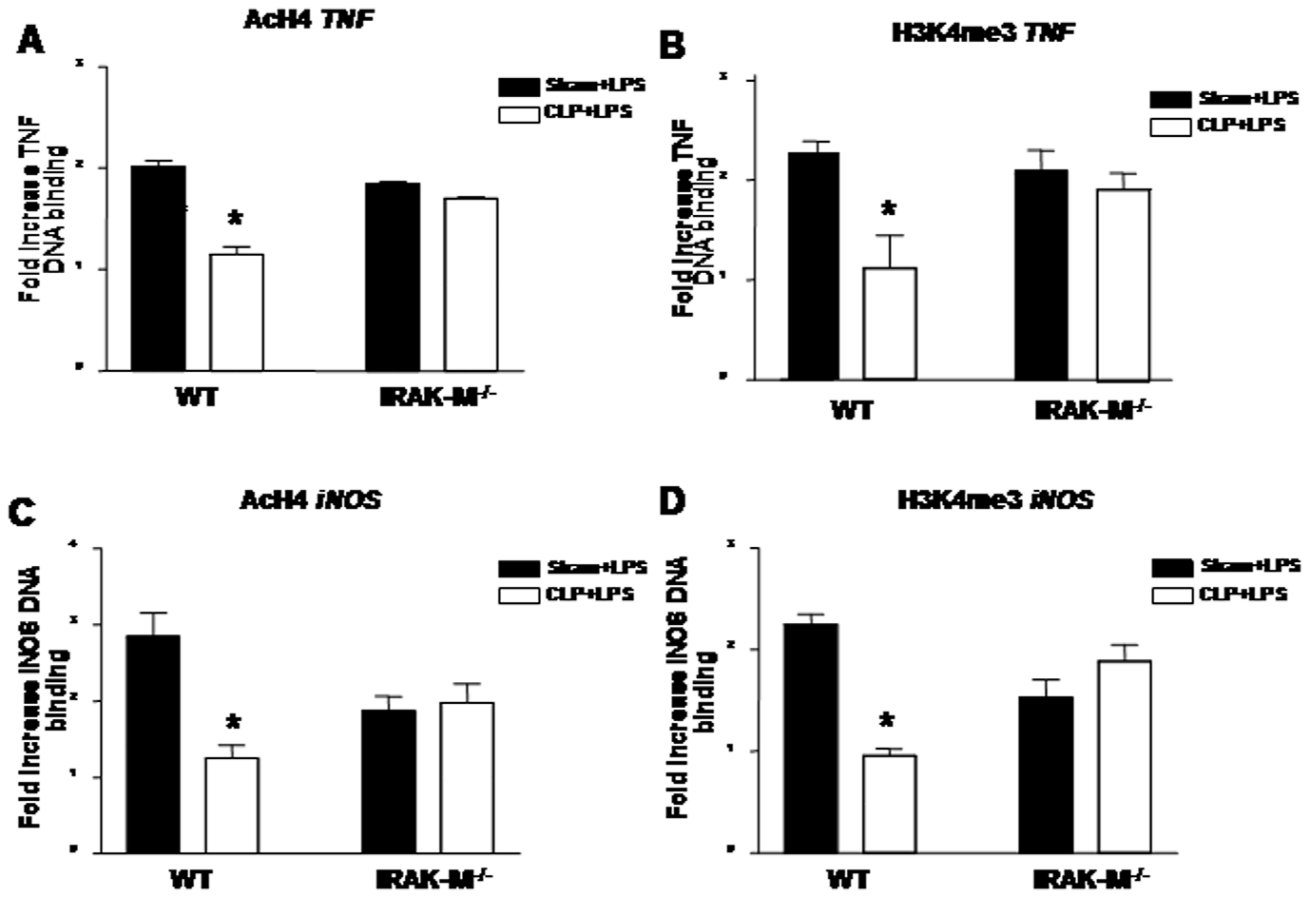
Histone modifications in PM isolated from WT and IRAK-M^−/−^ mice after sham surgery or CLP. Level of specific histone acetylation (AcH4) and trimethylation (H3K4me3) at the TNF-α promoter (A and B) and iNOS promoter (C and D) 2 hrs post LPS, as determined by ChIP analysis and expressed as fold increase over unstimulated sham cells. Results shown represent mean of 3 separate experiments, *p<0.05 as compared to WT sham PM treated with LPS.

We next assessed whether or not IRAK-M mediated the alterations in selected histone PTMs during sepsis. Treatment of WT PM with LPS ex vivo resulted in an increase in both AcH4 and H3K4me3 binding to the TNF and iNOS promoters in PM isolated from sham mice, whereas levels of AcH4 and H3K4me3 were diminished in PM from WT mice isolated 24 hrs post CLP (Figure 6). In contrast, the levels of AcH4 and H3K4me3 were largely maintained in LPS treated PM isolated from IRAK-M^−/−^ mice 24 hrs post CLP as compared to similarly-treated sham PM from IRAK-M^−/−^ animals. Likewise, binding of RNA polymerase to the TNF-α promoter was significantly reduced in WT PM 24 hrs after CLP when compared LPS-treated WT sham PM (Figure S1), whereas RNA polymerase II promoter binding was only minimally reduced in IRAK-M^−/−^ PM post CLP as compared to sham IRAK-M^−/−^ PM.

### IRAK-M regulates the expression of HDAC-2 during sepsis

**Figure 7:**
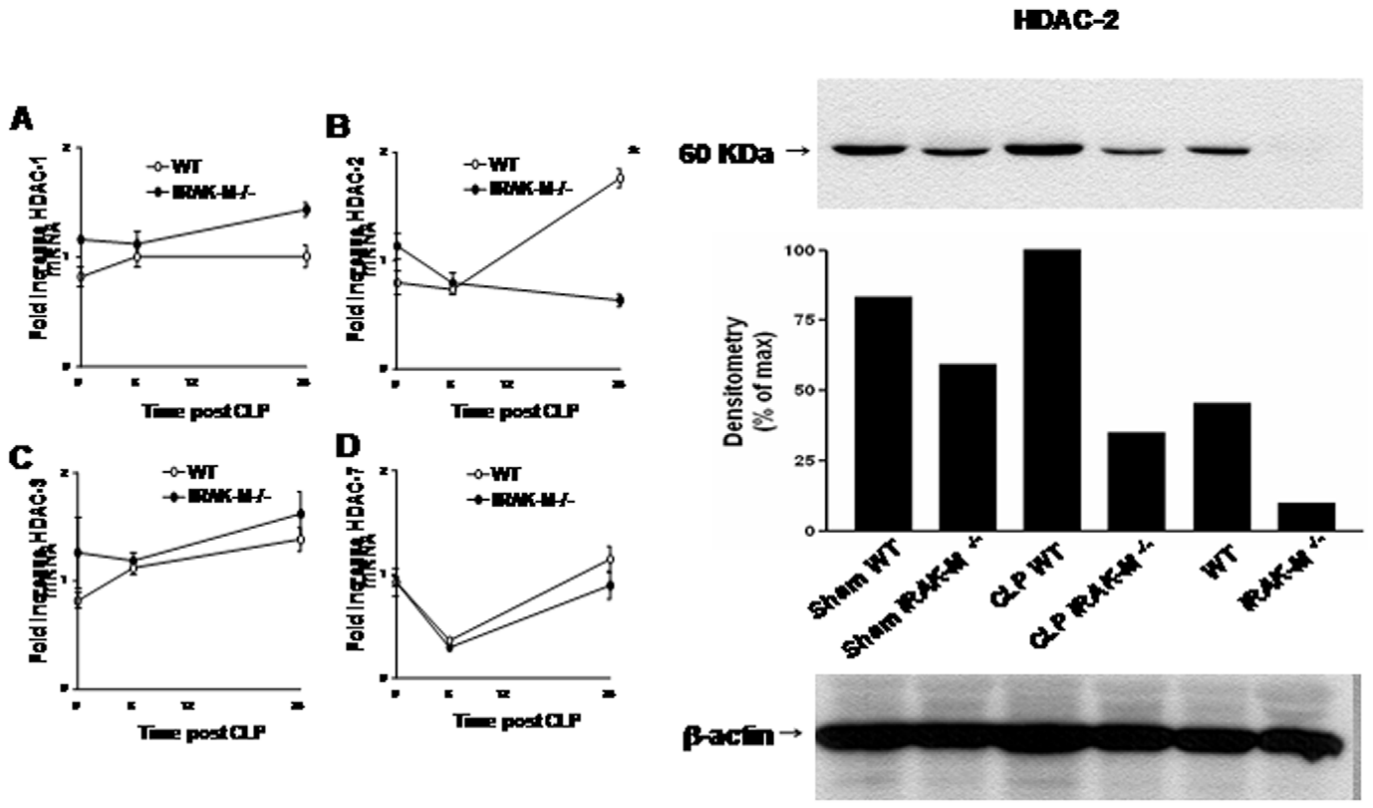
Induction of HDAC mRNA (left panel and protein (right panel) by PM isolated from WT and IRAK-M^−/−^ mice after CLP or sham surgery. A. HDAC-1 mRNA expression; B. HDAC-2 mRNA expression; C. HDAC-3 mRNA expression; D. HDAC-7 mRNA expression. Each time point  =  pooled cells from 3 animals per group, *p<0.05 as compared to CLP/IRAK-M^−/−^ group. Right upper panel is Western blot showing HDAC-2 protein expression 24 hrs post sham surgery, CLP, or no surgery. Middle panel is densitometry, lower panel β-actin. Each lane is pooled cells from 3 animals per condition.

We previously showed that HDAC activity is increased in PM post sepsis (Figure 3), and that IRAK-M regulated the magnitude of H4 acetylation. To determine if IRAK-M regulated the expression of Class 1 HDACs in sepsis, we isolated PM from WT and IRAK-M^−/−^ mice at 0, 6, and 24 hrs post sham surgery or CLP and assessed for mRNA expression of HDAC-1, -2, -3, and -7. As compared to baseline, no significant changes in the mRNA expression of HDACs -1, -3, or -7 were noted post sepsis, and levels of expression were similar in PM from WT and IRAK-M^−/−^ mice. In contrast, a nearly 2-fold increase in levels of HDAC-2 mRNA were noted in WT PM 24 hrs post CLP. Importantly, no induction of HDAC-2 was noted in IRAK-M deficient PM. To confirm differences in HDAC-2 at the protein level, we performed Western blot analysis for HDAC-2 in PM 24 hrs post sham-surgery, CLP, or no treatment. As compared to WT controls, HDAC-2 levels were increased in PM isolated from WT sham and especially CLP mice (Figure 7, left panel). Under all conditions, HDAC-2 protein levels were lower in PM isolated from IRAK-M^−/−^ mice than that observed in similarly treated WT PM.

## Discussion

Our findings indicate that abdominal sepsis results in marked defects in the ability to express important innate genes. The phenotype of lung macrophages isolated from septic mice is analogous to that observed in endotoxin tolerized monocyte/macrophages, whereby exposure to LPS or other PAMPs dampen the release of inflammatory mediators upon subsequent PAMP re-challenge [11], [26], [27]. We observed suppression of several inflammatory genes, including activating cytokines and iNOS. Similar to endotoxin-tolerized macrophages, we found these defects to be reversible, and noted that some host defense genes (e.g. Lcn2) were non-tolerizable and in fact induced in response to abdominal sepsis. The selective inhibition of certain but not all genes is consistent with a reprogrammed macrophage phenotype rather than a global state of deactivation [11].

The molecular mechanisms accounting for sepsis-induced macrophage reprogramming are almost certainly multiple. We noted considerable induction of the TLR signaling inhibitor IRAK-M in PM during the evolution of sepsis. The maximal expression of this protein was coincident with the time of most profound blunting of LPS-induced cytokine responses. Moreover, the tolerance phenotype observed in septic PM was largely reversed in PM isolated from IRAK-M^−/−^ mice, implicating this protein as a major mediator of sepsis-induced tolerance. IRAK-M has previously been shown to mediate macrophage tolerance to LPS and the TLR2 agonist peptidoglycan in-vitro, as well as tolerance responses in non-hematopoetic cells such as biliary epithelial cells [14], [18]. Interestingly, the induction of IRAK-M during the septic response appears to be MyD88-dependent, indicating that either TLR or IL-1 signaling pathways induce IRAK-M in an autoregulatory fashion to negatively regulate these inflammatory cascades. IRAK-M appears to be a relevant regulatory molecule in human sepsis, as the expression of IRAK-M is enhanced in blood monocytes isolated from patients with sepsis, and levels in pediatric sepsis patients directly correlate with adverse clinical outcomes in this patient population [11], [17].

Our findings indicate that sepsis resulted in remodeling of chromatin that may provide a molecular basis for reduced expression of selected innate genes. Specifically, we found reduced acetylation of histone 4 and trimethylation of lysine 4 of histone 3. Because these epigenetic marks mediate activation of selected genes, reduced acetylation/methylation would function to extinguish gene expression by limiting access of critical promoter regions to transcription factors, coactivators, and RNA polymerase. Studies performed in endotoxin-tolerized macrophages using HDAC and demethylase inhibitors (TSA and paragyline, respectively) suggest that reduced acetylation/methylation in LPS tolerance is due to more rapid deacetylation and dimethylation of histones rather than impairment in initial LPS-induced histone acetylation and methylation [26]. Increased acetylation of H4 post treatment with TSA, particularly in PM from CLP mice, supports enhanced HDAC activity in sepsis. Interestingly, the reduction in AcH4 and H3K4me3 observed in stimulated CLP PM was not observed in PM isolated from IRAK-M^−/−^ mice. A possible explanation for this finding is that IRAK-M might regulate the expression of TLR-induced HDACs or demethylases. To this end, we found a selective increase in the expression of HDAC-2 mRNA and protein in CLP PM isolated from WT but not IRAK-M^−/−^ mice LPS has previously been shown to regulate the expression of certain HDACs, although changes in HDAC expression during sepsis have not been defined [19]. Our data suggests that IRAK-M may either directly or indirectly induce HDAC-2 production. The mechanism accounting for this effect is unknown but is currently being investigated.

An alternative form of chromatin remodeling leading to gene silencing is methylation of cysteine residues within CpG islands in the promoter region [22]. While CpG methylation can mediate more sustained suppression of certain genes, the promoters of several of the inflammatory genes suppressed in sepsis, including TNF-α, IL-6, IL-12 p40, and iNOS, contain few CpG motifs and are therefore less susceptible to this form of epigenetic regulation.

In summary, our study shows for the first time epigenetic changes in lung macrophages during sepsis that mimic that observed in LPS-tolerized macrophages. IRAK-M plays a critical role in mediating macrophage reprogramming during sepsis, which may be due, in part, to regulation of chromatin remodeling that controls inflammatory gene transcription. Targeting enzymes/proteins that modify these epigenetic changes may be a promising approach to correcting dysregulated innate responses in sepsis.

## Supporting Information

Figure S1 RNA polymerase II promoter binding in PM isolated from WT and IRAK-M−/− mice after sham surgery or CLP. Shown is binding to the TNF promoter (A) and the iNOS promoter (B) 2 hrs post LPS, and expressed as fold increase over resting sham PM. Results shown represent mean of 3 separate experiments, *p<0.05 as compared to WT sham PM treated with LPS.
(0.04 MB TIF)
