# Supplementary figures and images for: IRAK-M Regulates Chromatin Remodeling in Lung Macrophages during Experimental Sepsis
Source: PLoS One. 2010 Jun 16;5(6):e11145. doi: 10.1371/journal.pone.0011145 (PMC2886833; doi:10.1371/journal.pone.0011145)

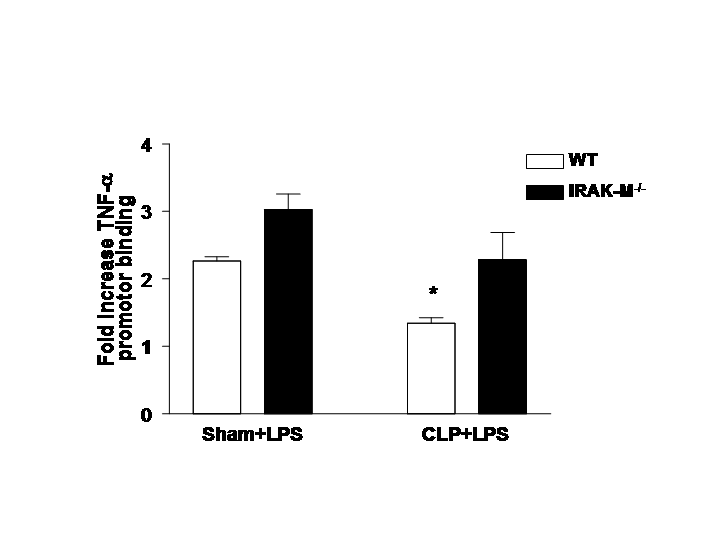

Supplement: Figure S1 — RNA polymerase II promoter binding in PM isolated from WT and IRAK-M−/− mice after sham surgery or CLP. Shown is binding to the TNF promoter (A) and the iNOS promoter (B) 2 hrs post LPS, and expressed as fold increase over resting sham PM. Results shown represent mean of 3 separate experiments, *p<0.05 as compared to WT sham PM treated with LPS. (0.04 MB TIF) [file pone.0011145.s001.tif]
